# Domain-Agnostic Representation of Side-Channels

**DOI:** 10.3390/e26080684

**Published:** 2024-08-13

**Authors:** Aaron Spence, Shaun Bangay

**Affiliations:** School of Information Technology, Deakin University, Geelong 3216, Australia; shaun.bangay@deakin.edu.au

**Keywords:** side-channel sensing, target information, medical diagnostics, human–computer interaction, cybersecurity, side-channel, framework, domain-agnostic

## Abstract

Side channels are unintended pathways within target systems that leak internal target information. Side-channel sensing (SCS) is the process of exploiting side channels to extract embedded target information. SCS is well established within the cybersecurity (CYB) domain, and has recently been proposed for medical diagnostics and monitoring (MDM). Remaining unrecognised is its applicability to human–computer interaction (HCI), among other domains (Misc). This article analyses literature demonstrating SCS examples across the MDM, HCI, Misc, and CYB domains. Despite their diversity, established fields of advanced sensing and signal processing underlie each example, enabling the unification of these currently otherwise isolated domains. Identified themes are collating under a proposed domain-agnostic SCS framework. This SCS framework enables a formalised and *systematic* approach to studying, detecting, and exploiting of side channels both within and between domains. Opportunities exist for modelling SCS as data structures, allowing for computation irrespective of domain. Future methodologies can take such data structures to enable cross- and intra-domain transferability of extraction techniques, perform side-channel leakage detection, and discover new side channels within target systems.

## 1. Introduction

Systems of all kinds generate a cacophony of observable auxiliary signals as a byproduct of their normal operation. CPUs consume electricity, WiFi signals reflect off surrounding objects, and heartbeats cause vibrations. Such signals are often disregarded as noise; however, extractable and valuable information is embedded within these noisy signals [1,2]. Such an indirect and often unexpected pathway to target information contained within or generated by a target system is known as a *side channel*. We define the process of exploiting side-channels as side-channel sensing (**SCS**): the utilisation of available sensor data in a non-trivial way to acquire previously unknown, hidden or unused target information from target systems.

Traditionally established within the cybersecurity (**CYB**) domain and its respective cyber–physical systems [1,3,4,5], emerging works demonstrate the existence and applicability of SCS in other domains. This includes the human body for medical diagnostics and monitoring (**MDM**) [1,6] and pairing of humans with cyber–physical systems for human–computer interactions (**HCI**) [2,7]. An additional domain category, termed miscellaneous (**Misc**), represents examples of side channels within civil, environmental, and other systems that lack the critical mass for grouping under one domain [2]. As with their CYB target system counterparts, side channels provide indirect and non-obvious access to internal target information.

The identified HCI and Misc domains apply SCS, albeit implicitly, without formal methodologies and with solutions presented in isolation. Conversely, the CYB domain explicitly recognises side channels with formal methodologies. However, their developments are entirely siloed from the other domains, designed solely for their respective cyber–physical target systems (e.g., electronic-based computational systems) and bespoke designs (e.g., using x86 instruction sets [8]) [1,2]. Recent works have formalised SCS within MDM and demonstrated how MDM and CYB can be unified [1]; however, HCI and Misc are omitted. Thus, a systematic SCS framework that is domain-agnostic is needed. This would enable a more formalised and *systematic* approach to studying side channels, detecting their presence and exploiting them both within and between domains.

Furthermore, such an SCS framework would allow for the development of data structures that capture real-world examples of SCS, irrespective of domain. Such data structures could be utilised computationally to model side-channel leakage or discover new instances [9,10]. Such discoveries have ramifications for target systems, such as revealing security breaches and data leaks [9,11]. or developing novel sensing solutions for acquiring target information such as a person’s heart rate [1]. We further hypothesise that a unified representation would promote the transferability of SCS extraction techniques and approaches both between and within domains. For example, Lange et al. [6] demonstrated the explicit migration of SCS extraction techniques. They utilised techniques derived from CYB against electronic-based computational target systems (e.g., PCs) to infer a person’s PIN via their electroencephalogram (EEG), a MDM solution against a biological target system (e.g., a person’s mind).

This article presents a theorised solution to the identified gap in literature, namely, that SCS is applicable to multiple but isolated domains. The objectives of this article are as follows:

1. To collate and systematically review the literature demonstrating SCS across the MDM, HCI, Misc, and CYB categories in order to derive their shared fundamental principles.
2. To capture each domain’s approach to SCS under a standardised set of terms that encompass all components of the SCS process, from side-channel leakage to target information acquisition, via extraction techniques. Such defined terms must be applicable for adequately describing SCS across all domains, i.e., be domain-agnostic.

The contributions of this article are:

1. To formally establish that utilisation of SCS exists within HCI and Misc domains, albeit implicitly.
2. Establishment of a domain-agnostic SCS framework that provides unified definitions of the SCS process and demonstrate its applicability to the MDM, HCI, Misc, and CYB domains.
3. Discussion of enabled opportunities: the cross- and intra-domain transferability of SCS extraction techniques, and avenues for SCS data structure representation for side-channel leakage detection and side-channel discovery methodologies.

This review begins with a standardised set of terms, each describing a component of the SCS process applicable to all domains. Literature demonstrating SCS across identified domains is collated and analysed in respect to each SCS component (Section 2). Shared fundamental themes between the domains are extracted, resulting in a defined domain-agnostic SCS framework that sufficient describes the entire SCS process and corresponds with real-world observations. The review concludes with discussion of opportunities arising from the establishment of a domain-agnostic SCS framework, including avenues for future work (Section 3). The content of this review is based on the corresponding author’s PhD thesis [12].

## 2. SCS Across Domains

**Figure 1:**
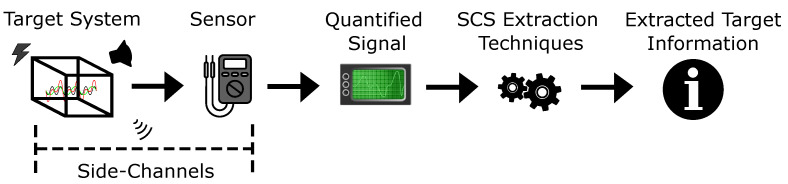
An illustration of the domain-agnostic SCS process.

The SCS process involves the quantification by sensors of signals traversing unexpected indirect pathways (i.e., side channels) within target systems. Captured signals are analyzed via extraction techniques to acquire embedded target information (Figure 1) [1]. For instance, a HCI solution capturing hand gestures visually via a camera is too direct and ‘obvious’, whereas capture via acoustics [13] is less obvious and non-trivial (i.e., a side channel). A SCS framework applicable to and encompassing target systems from any domain does not currently exist. The HCI and Misc domains apply SCS, albeit implicitly, without formal methodologies and with each solution presented in isolation; conversely, the CYB domain explicitly recognises side-channels with formal methodologies. However, their developments are entirely siloed from each other [1,2]. Recent works formalised SCS within MDM and consolidated it with CYB [1]. This section analyses literature demonstrating the definition of SCS across these domains while isolating the target systems involved along with the utilised techniques and mindsets.

Searching for relevant keywords (i.e., side channels, side-channel attacks, covert channels) is ineffective for MDM, HCI, and Misc because the term is not recognised in the respective literature [2]. Instead, articles were seeded from related terms (i.e., sensing, sensors, diagnostics, human–computer interaction, smartphones, wearables, gold standard). Additional papers were identified through online articles reporting on unexpected or non-trivial sensing opportunities. Analysis involved identifying shared principles around how SCS is implemented within each domain. Of interest was how signals traverse, their properties (e.g., some signals may propagate over distance), how they are sensed, and how they can be capitalised on to achieve SCS. Questions arise such as what sensing devices are used and why, and whether solutions are passive or invasive. A synthesis of the literature reveals themes within the respective domains and contributes towards defining SCS in a domain-agnostic way. Despite their diversity, we expect that they share underlying themes in their SCS processes. The result is a SCS framework unifying the extracted themes, providing definition and formalisation to the field of SCS in a way that is applicable to all domains.

### 2.1. SCS Framework

Current SCS-related literature either lacks consistent terminology and definitions or uses a disparate collection constrained to a specific domain and context [1]. We begin by synthesising a set of standardised terms which (i) encompasses all components of the SCS process, and (ii) is applicable to SCS across all domains. Table 1 illustrates the classification of terms used in this review. Together, these terms describe the entire multi-stage SCS process (Figure 1). We derive the commonalities and differences between domains in respect of their employment of SCS under these unifying terms.

#### 2.1.1. Target Information

Target information is the desired information, as distinguished from other information or noise present within the target system. It exists embedded within signals contained/generated within a target system that leak to sites accessible by a sensor.

**CYB**  focuses on target information within electronic-based computational devices. These may be secured cryptographically [15,16,17,18,19], but can also include personal information that is leaked unintentionally, such as media viewing [20], web-browsing habits [21,22,23], and information communicated or generated by devices such as printers [24,25,26]. In rare cases, non-electronic target information is included within the CYB-related literature, such as PIN codes stored in a person’s brain being extracted through analysis of EEG readings [6,7].
**MDM**  quantifies physiological or physical parameters within the human body (MDM’s target system). Examples include heart rate [27,28,29,30,31], breathing rate [27,32], chemical concentrations (e.g., oxygen) within the bloodstream [33,34], and tremors [35,36]. Conditions with associated physiological manifestations (including psychological conditions with physiological biomarkers, such as cognitive load via pupillary response [37]) also have the potential to be accessible via side channels.
**HCI**  infers the state or intention of humans (i.e., the user) expressed through physiological parameters. This includes gestures initiated by the eyes [38], jaw [39,40,41], tongue [39], hands [13,42], and fingers [43,44]. Such gestures (e.g., moving the eyes from left to right [38] or scratching a surface in an ‘X’ formation [42]) are sensed (e.g., by a smartphone) and the user’s intention is inferred. Unlike other domains, information is part of a feedback loop in which the response from the device loops back to the human, who can then adapt their actions to manipulate the sensing process. For example, touching the flashlight to reduce the light level detected by the camera to infer pressure [43].
**Misc**  is diverse in its target information; it is no longer just a read-only quantity, but can be injected for communication [45], control [46] or to support other forms of sensing [47,48]. The diversity of application areas allows for categorisation of classes of target information:
  - **Mundane but Convenient:** While potholes [49] are easy to identify, augmenting an existing process, in this case taxis, provides data for minimal extra investment. Direct cross-technology communication [50] saves on deploying additional networking nodes.
  - **Unexpected:** Target information is well hidden and not previously used, such as geolocation from light flicker [51], screen content through audio leaked in a phone call [52], audio recovered from video [53], or human presence through WiFi signal strength [54,55,56].
  - **Abstract:** Target information that is not normally directly acquired by sensors but can be inferred from one or more channels, such as driver aggression [57] or software identification [58].

Target information can be digital information (CYB), a signal associated with some physical process (MDM), an abstraction such as the representation of a state (CYB, Misc), or an intention to perform a particular action (HCI). Opportunities exist to extract target information in any of these forms. In certain scenarios, partial recovery of target information may still provide value, perhaps by revealing insight into a particular execution path (CYB) or enabling a diagnosis (MDM).

#### 2.1.2. Target Systems

A target system is a bounded physical system that contains or generates target information. Understanding its internal operations can reveal the existence of target information as well as the structure of the side channel and its properties.

**CYB**  typically ‘attacks’ physical and electronics-based systems such as smart cards [19,59], FPGAs, microcontrollers, embedded devices, PCs [16,17,60], medical devices [61], smartphones [4,14,62], printers (traditional and 3D) [24,25], TVs [20], displays [6,7], and keyboards [63]. Exploitation of specific sensors rather an entire system can also be a side-channel source through signal transformations and injection of signals/values into the signal measured by the sensor [5]. These devices share fundamental exploitable properties such as power consumption, clock rates, acoustic noise generation, and electromagnetic radiation. Consequently, all systems that share a demonstrated exploitable property are vulnerable to SCS [2].
**MDM**  focuses on the human body: an inherently complex and interconnected system. Consequently, it is a rich source of side channels, and can be viewed as an amalgamation of components (or subsystems) in a number of ways. For example, it can be segmented based on the location of components such as the back, thorax, and abdomen [64]. Each in turn is comprised of subcomponents such as the musculoskeletal components of the back (vertebrae, scapula, vertebral column, and pelvic bone) [64]. An alternative view is of its major systems (e.g., the nervous system) [65]. Spatial coherence supports tracking of the physical paths followed by target information through neighbouring components. A condition’s cause inhabiting one component may manifest entirely elsewhere, creating opportunities for information leakage [1]. The human body’s interconnectivity is a fundamental principle that should be considered when approaching SCS for MDM.
**HCI**  interconnects two distinct target systems, namely, biological systems (e.g., a human’s intentions and intended movements) and electronics-based systems. HCI solutions use the electronic systems to ‘sense’ an appropriate side-channel signal identified within the biological system. The signal is input to a computer which contains an actuation element, such as a display, which feeds a response of the sensed signal back to the human. Unlike MDM, where the human is a passive generator of signals, in HCI signals are actively generated through intentional gestures [13].
**Misc**  may exhibit a selection bias in which it is likely to identify more extreme system structures. Examples range from using generic smartphone sensors to categorise driver behaviour [57], exploiting the existing complexity of a taxi network [49], and more complex interactions. Insights into the system configuration, such as wall position and router placement [56], are parameters used to support signal processing. Compared to previous domains, these target systems place less emphasis on the physical co-location of system components. Existing active elements in the target system are exploited by measuring changes in reflected signals, for instance to detect movement [54,55] or by using potholes to induce pain levels [66]. New elements are added to trigger a side-channel response [48]. Examples in the Misc category demonstrate greater diversity in terms of the system components exploited or added compared to those of the other domains. As illustrated by the potato chip bag microphone in [53], any system component (specifically in addition to electronics or local active elements) can be a relevant part of the target system. The boundary of target systems is also less well defined ( versus, e.g., a smartphone), covering large areas such as a country’s electrical grid [51], or a road network [49].

A target system consists of its components and their interconnections. System structures associated with side channels have long paths (sequences of components) which increase leakage opportunities [1,9,10]. The interconnections between components allow information to be mixed and distributed to different destinations. Information can be retrieved by manipulating components to inject signals, trigger actions, or generate signals that provide insight into internal operations, such as reflected signals from a WiFi router [56].

CYB components are well documented but tend to actively resist attempts to extricate target information. In contrast, the biological components in MDM applications are modelled empirically. Design documentation is not provided, with often complex interactions between components. MDM systems also support multiple valid classifications for components based on various structural or logical decompositions. As a result, MDM solutions have not been approached from a system viewpoint but rather by replicating gold standard output [1,33,67]. HCI scenarios involve a fusion of both engineered and biological elements, with the addition of feedback loops [68]. The exception cases represented in Misc often involve target systems distributed over (sometimes significant) geographical areas [49,51,57].

#### 2.1.3. Side Channels

Side channels are the pathways that a leaked signal traverses while containing embedded target information. They allow target information to be obtained indirectly or via novel means through signal transformations or modifications (see ‘Side-Channel Properties’ in Section 2.1.4). Target information is often ‘leaked’ from its source components to adjacent components within the target system (perhaps as heat, sound, etc.), often unintentionally or without being noticed. It is presumed that the target information cannot be directly sensed, either because it is actively concealed, inaccessible, expensive, or non-ideal. A side-channel allows these constraints to be bypassed.

**CYB**  capitalises on a property of side channels whereby the signal embedded within is transformed and modified during traversal. For example, target information originating from a website could be sent as internet traffic, stored in a text file, and then printed to paper. Target information manifests physically as audio produced by dot-matrix printers [24], vibrations within 3D printers [25], or from observing the magnetic field of a laser printer [26]. Side channels do need to ultimately derive from target information. For example, when exploiting electromagnetic radiation for EDCH encryption algorithm key extraction [16], the side channel results from the mutual information between the internal processing of the EDCH decryption and the electromagnetic radiation emitted by the device. Multiple side channels can be viable for a single target system (e.g., PCs [15,69,70,71]); these may be exploited either individually or concurrently (e.g., sensor fusion) [25].
**MDM**  exploits biomarkers, which are quantifiable signals indicative of normal biological processes [72]. Biomarkers may exist internally or externally; thus, their observability varies. When internal, direct measurement with ubiquitous, cheap, and available sensors becomes non-trivial, with reliance on channels where the target information travels to a sensing site being more accessible. For example, dehydration has an externally available biomarker in the level of pH in sweat [73,74], which is quantifiable via colorimetric strips and can be analysed through a smartphone camera [75]. Established gold standard diagnostic devices rely on proven biomarkers. SCS offers the potential to exploit them in new ways or even discover new ones.
**HCI**  exploits the way in which information ‘leaks’ along side channels, with SCS solutions existing for the human and/or computer components:
  - **Computer channels:** Because channels are not deliberately protected (e.g., encrypted), HCI deals with primary channels used in an expected or non-trivial way or via existing or introduced side-channels (for example, sensing pressure through the interaction between accelerometer and vibration generated by the motor built into a smartphone) [44].
  - **Human channels:** Channels exploited in HCI are those that carry information to more accessible or convenient external locations, such as using electrodes placed within the earlobe to detect eye movement [38].
  - **Feedback loops:** Both humans and computers can deliberately generate and respond to signals produced by the other component [68,76].
**Misc**  involves long pathways from the target information to the sensor [51], and includes signal transformations such as video to sound [53] and separation of mixtures of signals [77]. Missing scenarios include those where the side channel is predominantly virtual (information), as physical interactions are favoured. As such, a side channel exists as one of a number of signals mixed into physical signals, such as light flicker in a video [51]. Otherwise, the side channel may be fragmented across multiple signals, requiring sensors for all signal types (such as acceleration and rotation to recover driving behaviour [57], or multiple sensor elements using the same signal to enhance recovery in a rolling shutter with individual image elements to recover a high frequency signal) [53].

Side channels exist in target systems from any domain, respresenting a key insight expressed and explored in this research. Side channels result from entanglement operations within the target system. They allow one signal to be detected by sensing it when mixed in with another. Correlation is one approach used by all domains for identifying side-channel candidates, and is usable even when the causal relationship is not well understood [1]. CYB treats side channels as containing hidden information that needs to be recovered through cunning (i.e., outmanoeuvring defences or obstacles) [69,70]. In contrast, MDM exploits surprising pathways with embedded target information. HCI side channels are not deliberately hidden, but exist due to difficulties in directly sensing the target information. Feedback is one way of achieving the tuning of system parameters and sensor placement required to achieve SCS [68,76]. While electronic and physical/biological channels represent information differently, analogous operations are possible in both. In certain cases, access to one side channel improves opportunities to extract another related channel.

#### 2.1.4. Side-Channel Properties

The following side-channel properties that denote the shared characteristics of all side-channels emerge irrespective of target system and domain [2]:

- **Determinism**: There is a reproducible, reliable correlation between internal operations and any quantified signals.
- **Multi-Stage Pathways**: A signal often traverses multiple stages along its pathway from internal interconnected components to its sensing site. This makes discovery of all paths non-trivial.

Understanding of side channels via their properties provides insights into how to extract their target information, and even into how to discover them. The key is *determinism*. A reliable correlation between the internal source and quantified output signals indicates a candidate side channel. This is the case irrespective of whether the mechanism enabling it is understood. Furthermore, all side channels are indirect by definition; therefore, the *multi-stage pathway* property is present in all candidate side-channels. The following additional side-channel properties reflect how signals are subject to a myriad of modifications during traversal of a side channel:

- **Signal Transformations**: Signals are transformative between states. In MDM for example, excessive bilirubin buildup in the bloodstream (a by-product of recycling red blood cells) manifests as a yellowish discolouration of the skin and sclera if subject to jaundice [67]. Transformations may be invoked via interference (perhaps intentionally by an observer), signal reflection/refraction, or due to obstructions. Additionally, they may occur both within and beyond the target system’s boundary. Signal transformations are capitalised on by all domains, either via custom sensors or, as is common in MDM and HCI, using existing sensors on a constrained platform (e.g., a smartphone).
- **Modulation Proportion**: How prominent the target information is within a side channel is determined by its proportion in relation to the signal in which it is embedded. A low proportion when mixed with other signals may make the target information difficult to detect.
- **Signal Mixing**: Target systems internally consist of interwoven channels in which their signals mix and collide. Target information that is not directly accessible may instead be detected when mixed with another signal. Alternatively, a signal external and alien to the side channel (or even the target system itself) could be injected and mixed into the side channel.
- **Multivariate**: A single target information source can have multiple associated side channels, increasing the number of available attack vectors.

**Figure 2:**
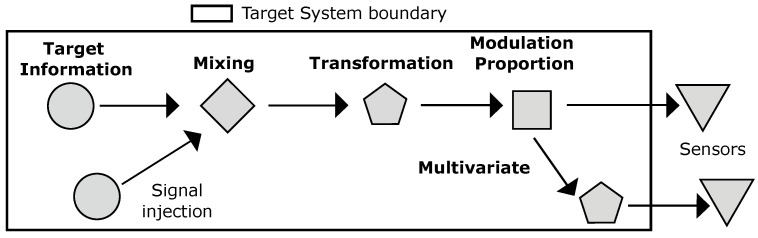
Side-channel properties describe the structure and behaviour of side channels and their embedded signals.

Such properties provide insight into the side-channel signal’s structure and embedded target information. The *multi-stage* nature of side-channels means that side channels emanate and traverse from their source in a myriad of ways, during which time side-channels are mixed, transformed, and vary in *modulation* (Figure 2). Consequently, multiple side channels may be plausible for the same target information. MDM is an example, with the human body’s interconnected nature promoting an array of side channels. For example, a single instance of target information such as the heart rate is accessible by traditional gold standards (ECG, stethoscope) as well as by “less obvious” side channels: within the ear canal [78], via chest and head movement [79,80,81,82], photoplethysmography by a camera view of the ear canal [83] or face [30,31,74], or monitoring of chest oscillations via WiFi (e.g., electromagnetic radiation) [32].

Identifying which properties are present and to what extent can dictate how a signal is quantified. For instance, signal transformations allows sensors to capture target information that is outside the intended sensing capability, for example, using a microphone to sense pressure variations within the lungs [33] or using speakers to operate as a microphone [71]. Transformations may enable unexpected and novel SCS solutions that were not previously possible or that improve on existing solutions. The presence of *multivariance* indicates that a single target information may have multiple viable sensing sites, with some being more viable and others potentially impossible.

#### 2.1.5. Information Parameters

‘Information’ is a core theme throughout SCS solutions, and is prominent across the entire SCS process. From an information theory perspective, Shannon [84] formalised the representation and quantifying of information in respect of its communication over a channel to a receiver. Similarly, SCS involves the communication of target information in the form of a signal over a channel in order to be received by a sensor and deconstructed. The view that information is quantifiable and communicated is adopted in CYB with the side-channel vulnerability factor (SVF), a method to quantify the *amount* of information that is present or leaked via side channels [85]. Such a method is valuable for assessing a target system’s level of exposure to unauthorised access. The different domains demonstrate that it is need not necessary to quantify the *amount* of target information being communicated in order to achieve SCS; instead, greater reliance is placed on the side-channel property of *determinism* (Section 2.1.4).

The SCS examples define target information and signals in the physical sense, e.g., electromagnetic radiation. Each comes bundled with associated properties that describes that signal. For example, the associated properties of electromagnetic radiation include frequency, propagation, and refraction. Viewed together, the domains exhibit trends that go beyond physical modalities, for instance, solutions utilising *remote sensing* (tablet screen replication through leaked *electromagnetic radiation* [86] (CYB), lung health classification via *sound* [33] (MDM), gesture recognition via *sound* through a surface [42] (HCI), remote sensing of human movement via WiFi (i.e., *electromagnetic radiation*) [56] (Misc)). These examples, varying by domain and target system, are connected not by the utilised physical modalities (i.e., electromagnetic radiation and sound) but by the shared properties of these modalities. Fundamental to this is information that *propagates* over distance and/or through materials with measurable *frequencies*. Such descriptions enable side channels to be understood in terms of their *information parameters*. This provides an abstracted view of side channels and a foundational step towards unifying SCS across domains. SCS examples that appear disconnected may actually share common information parameters in their sensed signals and/or utilised SCS extraction techniques.

#### 2.1.6. Sensors

Sensors quantify the information parameters of the signal at a specific location along the side-channel.

**CYB**  selects sensors most appropriate for the signal in question (e.g., copper coils for electromagnetic radiation [87]). Measurement considers the reduction of extraneous information sources, the placement of sensors, and concurrent internal processes within a target system [14]. CYB recognises that noise is potentially the *source* of side channels. Trial and error is common for refining measurements, such as finding the optimal location to sense electromagnetic radiation from a PC [16].
**MDM**  utilises sensors along a spectrum as per its context specific suitability, as described by Spence and Bangay [1]:
  - **Stand-alone sensors:** Large variety, small size, ubiquitous, and easily embedded into devices [88].
  - **Wearables:** Ubiquitous, real-time continuous monitoring, with direct or near-body contact. Customisable via additional sensors [89] and attachments [90,91].
  - **Smartphones:** Ubiquitous, real-time continuous monitoring, in proximity to the body, built-in processing capabilities [27]. Ability to customise or enhance sensing capabilities with attachments [92,93].
  - **Wearables/smartphones with remote server:** Increased processing capabilities for analysis, opportunities for data aggregation from multiple sources [89,90].
**HCI**  adopts a large array of signal types. This is due to HCI’s affinity for prototyping and its focus on two distinct target systems, one electronic-based and the other biological-based. Emphasis is placed on consideration of the information parameters of the quantified signals and how they can be best exploited. Acoustics propagate along or through materials [42] and over distance through empty space [13], both of which enable detection of user input and movements at distance. Sensors can be placed at varied and multiple locations and quantify signals locally or remotely depending on the information parameters being exploited.
**Misc**  tends to deal with signals originating from physical processes. This can be exploited by deliberately triggering a signal change [45,48]. Sensors range from those already available in a standard smartphone [57] to customised rigs that adapt samples for better sensing (e.g., by staining samples to highlight allergens [94]) or actively injecting signals to trigger side channels [48]. Human detection using WiFi progresses from using customised hardware to using existing installations only [54,55,56].

Sensing in CYB and HCI is achieved with custom sensors deliberately chosen as per the information parameters in focus. Smartphones are commonly used in MDM and HCI because they are ubiquitous and portable with an array of embedded sensors. These factors allow for extended data collection, replacing brief access to high quality but expensive devices. Interesting sensors result from repurposing existing sensors, such as a lensless microscope produced by projecting holographic interference patterns onto a smartphone camera [95]. Examples exist where the same sensor (particularly on smartphones) is used for diverse purposes. In this way, SCS solutions can achieve the same outcome using very different pathways, for instance, through alternative versions of the same gold standard in MDM [1] or the CYB examples that identify screen content through power consumption, audio signals, or electromagnetic radiation. Use of various standalone sensors often indicates research that is in the prototyping stages, representing an effective strategy when approaching the non-obvious nature of side channels. Notably, sensor placement is as crucial as which sensor is used and how [96,97].

Sensing is enhanced by active sensing, which refers to injecting information to augment or cancel particular signals. This is most apparent for MDM when manipulating biometric samples, such as by staining blood samples [98]. HCI sensing tends to be active. The sensor’s output is fed back to the human participant, enabling them to adapt their response, thereby creating a feedback loop. The efficiency of sensing is tuned by manipulating sensor placement or by controlling parameters such as filter bands when processing the data. In Misc, sensor clusters such as collections of individual sensor elements in a camera are used collectively to compensate for the limited spatial and temporal resolution of any individual element.

#### 2.1.7. Methods and Extraction Techniques

Two related but distinct methodologies are employed to acquire target information. First, SCS methods detail *how* signals suspected of housing target information (i.e., side channels) are sensed. Second, SCS extraction techniques *extract* embedded target information from sensed signals [2]. Spence and Bangay [2] state that despite target system variance across domains (e.g., cyber–physical, biological, civil infrastructure), SCS methods can be abstracted so as to be applicable to all. They are classified as followed:

- **Invasive vs. Non-Invasive:** The target system is physically modified to provide (better) access to specific signals, or only originally accessible signals are sensed.
- **Active vs. Passive:** Control over the target system’s operations are exploited, perhaps by repeated execution of internal operations to trigger specific signals, or used to aid in the study of how internal operations work, such as the injection of particular input to measure the output signals and their behaviour.
- **Remote vs. Local:** The side channel’s *information parameters* (Section 2.1.5) define whether sensing can (and should) occur at a distance. Local measurements provide clearer signals, although this may not be possible depending on the level of access to the target system or the measurement intention (e.g., covert sensing).
- **Profiled vs. Non-Profiled:** With unimpeded access to the target system, a large number of measurements are collected to build a model of the determinism between its internal operations and sensed signals. Future measurements of this target system are analysed in the context of this profile.
- **Utilising existing third-party data:** Third-party collected data may already inadvertently house embedded target information.
- **Multivariate:** Sensor fusion techniques involve the collation of data from multiple sensed side channels output from the same target system.
- **Signal Injection:** Bespoke signals are injected into the target system to invoke observable or specific output signals. This also includes the intention to invoke faults (i.e., behaviour different to its original design) in order to create measurable outputs to learn more of the internal operations.
- **Software-based approaches to expand hardware:** Target systems that are not modifiable physically (per an *invasive* method) may limit available output signals. However, an array of sensors may already exist within the target system itself (e.g., a smartphone), and a *multivariate* approach can collate them to provide access to target information.
- **Repurposing Sensor:** Sensors used as actuators can often ‘sense’ target information beyond their original design intention, for example, the ability to recreate audio from visual recordings via a camera [53].

Similarly, Spence and Bangay [2] collated SCS extraction techniques abstracted so as to be applicable across domains:

- **Power analysis attacks:** Exploitation of deterministic correlations between internal operations and the output quantified signal, for example, a computational device’s power consumption or emitted electromagnetic radiation. Sufficient resolution in the quantified signal can reveal executed operations, including the execution sequence, from which target information can be inferred or reconstructed.
- **Information-theoretical analysis:** Treats signals as noisy, with target information muddled within. Encompasses signal processing techniques from information theory (e.g., Shannon’s entropy, Hamming weights), cryptanalysis, statistical analysis (e.g., maximum likelihood [99,100], correlation, or simple regressions), and transformations (e.g., FFTs).
- **Machine learning:** When large sensing datasets from side channels can be create or acquired, machine learning offers automated feature extraction stages for identifying how target information can be extracted.

The crux of our SCS research is that noise in a signal *may actually contain meaningful content (i.e., target information)*, thereby qualifying as a candidate side channel. The lack of a unified SCS framework results in solutions using ad hoc sequences of features, filters, and other individually tuned signal processing stages. Adoption of mindsets from varying domains represents an opportunity to reveal novel SCS extraction techniques; for example, one may ‘attack’ the human body for MDM purposes similarly to how cyber–physical systems are exploited within CYB [1].

SCS method pairings with applicable SCS extraction techniques are dependent on the defined constraints and intentions. CYB often has unrestricted access to its target systems; therefore, they are free to modify them with *signal injections* and *active* SCS methods or perform lengthy and repeated measurements to develop a *profile*. If covert sensing is an objective, *non-invasive* and *remote* methods are preferred. MDM and HCI are much more constrained. *Invasive* methods are unlikely to be ideal when performed on the human body, nor is there usually the option of performing enough measurements to build a *profile* of an individual. *Active* methods may be possible, such as requiring the patient/user to perform specific actions or movements in order to invoke desired signals (e.g., increasing the heart rate by running). HCI solutions are also more tolerant of errors; with the human as an active participant in a feedback loop, they are open to experimentation with *repurposing sensors* or substituting *software-based approaches*.

For all domains, information parameters (Section 2.1.5) are a driving factor, either limiting or enabling the applicable SCS methods and extraction techniques.

##### Reliance on Information Parameters

As defined, the ‘information’ represented by target information and side-channel signals is described in respect of its *information parameters* (Section 2.1.5). To operate, SCS extraction techniques take as input the required information parameters. These are then manipulated to extract the embedded target information. Adib and Katabi [56] used WiFi (electromagnetic radiation) from routers to track the movement of humans, even through walls, by employing signal processing and source separation extraction techniques. These techniques capitalised on the associated information parameters, frequency and duration, that WiFi propagates and reflects. Their extraction techniques are not *only* applicable to WiFi signals but to any side-channel signal from any domain or target system that shares the same information parameters.

#### 2.1.8. Summary

This analysis provides insight into how SCS is applied across domains. CYB is concerned with target information within electronic devices. Typical target information includes encryption keys [3], personal information such as media viewing [20] and web browsing habits [21,22,23], and eavesdropping, such as remote viewing of tablet screens [86]. MDM quantifies physical or physiological parameters within the human body (MDM’s target system) for the purpose of a diagnosis or monitoring of a medical condition. Except for psychological conditions (i.e., arising from or within the mind), all target information exists physically, where it may not be possible to measure directly without disturbing or invading the system [1]. HCI involves perception of the state or intention of the human. Such target information is expressed through physiological parameters such as gestures initiated by the eyes [38], jaw [39,40,41], tongue [39], hands [13,42], and fingers [43,44]. Unlike other domains, information does not just travel from human to computer. Instead, it is part of a feedback loop between computer and human, allowing both to adapt their actions in order to manipulate the sensing process. In such scenarios, the target system (the human) is an active participant in the SCS solution. Lastly, Misc demonstrates that SCS is relevant across more than the three primary domains, indicating that systems from any domain are susceptible to side channels and may be exploitable via new solutions.

**Figure 3:**
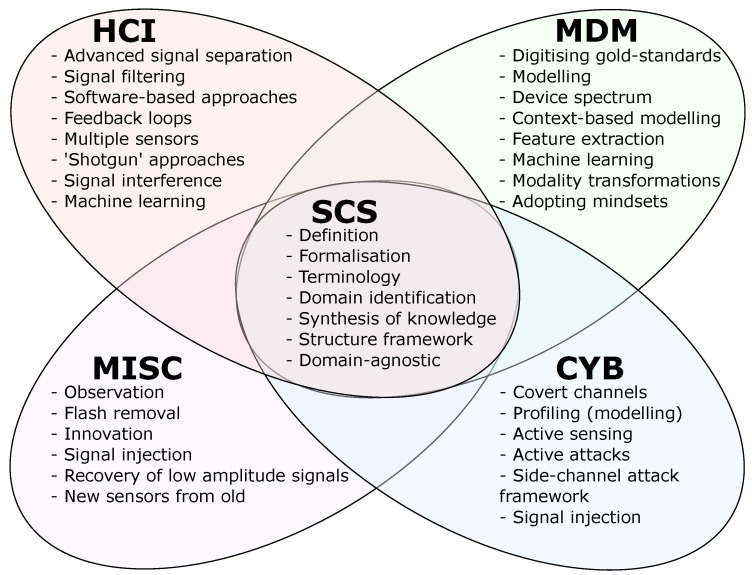
The SCS framework unifies the themes from each individual domain.

**Figure 4:**
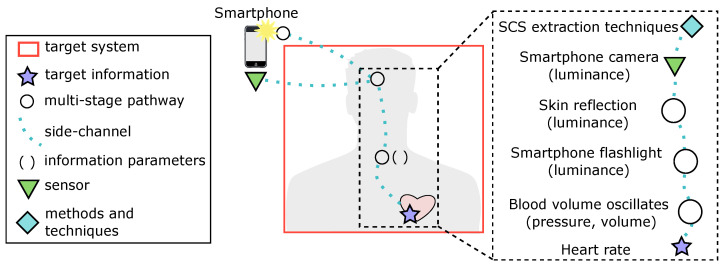
A side channel connecting heartbeats and skin colour variance. A smartphone quantifies this colour variance on a person’s forehead to infer heart rate (the target information). Side channels can be understood following the SCS framework components.

Despite this diversity, established fields of advanced sensing and signal processing underlie each example, enabling a unified view of otherwise isolated domains (Figure 3). Each term corresponds to an SCS component. Combined, they produce a SCS framework, providing an abstract and domain-agnostic unified definition of SCS irrespective of the specific environment and application. This enables side channels from any target system or domain to be understood abstractly and uniformly (Figure 4). By sufficiently describing the entire SCS process, the SCS framework components correspond with real-world observations. That is, target information embedded within a signal flowing from a target system (often unintentionally) along a side channel characterised by specific information parameters that capture its state and behaviour. These can then be exploited via an extraction technique to acquire the embedded target information.

## 3. Analysis and Discussion

The SCS framework is a theorised solution for unifying SCS demonstrated within multiple isolated domains. It establishes a unified, abstracted, and domain-agnostic definition of SCS across four domains. With this view, it is possible to derive the fundamentals of the SCS process and apply it to any scenario. Implementation of SCS is dependent on its environment, which is describable by the SCS framework. We recognise that all side channels exhibit a set of properties (Section 2.1.4). Together, these shape the identification and understanding of side channels. Within a target system, target information may be physically contained within or manifest from internal operations. Resulting signals that produce a deterministic correlation are prime candidates to be side channels. Traversing side-channel signals follow a multi-stage pathway during which they are likely to be modified or transformed due to side-channel properties involving signal transformations, modulation proportions, signal mixing, and multivariance. The signals will likely vary in the number of ’stages’ or modifications encountered in relation to their starting state. In theory, longer side-channel pathways have a higher chance of intertwining with other channels, which may introduce additional noise or enable novel sensing opportunities. Ultimately, side-channels are mutable, whether by their own volition or by external deliberate forces. For instance, certain SCS examples are only possible due to signal injections (e.g., flashlight, WiFi signal) to invoke the required signal-mixing side-channel property (Section 2.1.4).

A signal’s traversal ends once it is quantified by a sensor, either external or internal to the target system. The sensing site is dictated by (i) the information parameters involved, (ii) whether there is sufficient unrestricted access to the target system to allow for tampering, and/or (iii) covert or non-invasive sensing was an objective. Side-channel signals and their embedded target information are represented not by their physical modalities (e.g., electricity, sound) but by their *information parameters*, that is, the fundamental properties that describe them (e.g., frequency) (Section 2.1.4). A quantified signal needs to contain the information parameters that sufficiently denote the target information and can be subsequently processed via SCS extraction techniques [2].

This section explores opportunities enabled by defining a domain-agnostic SCS framework, each representing an avenue for future work.

### 3.1. Transferability of SCS Extraction Techniques

As each domain abides by the same SCS framework components, a hypothesis for intra-domain and cross-domain transferability emerges. The SCS framework has established that it is the *information parameters* that SCS extraction techniques require and exploit (Section 2.1.5). Thus, given that two side-channels share identical information parameters, a SCS extraction method demonstrated for one is applicable to the other. This statement applies irrespective of domain or target system, meaning that SCS methods and extraction techniques from all identified domains are applicable candidates within the others (Section 2.1.7).

Martinovic et al. [7] and Lange et al. [6] demonstrated an explicit transfer of CYB-derived SCS extraction techniques to a biological target system (i.e., MDM). They recognised that the brain is a bounded system with input and output signals; it ‘consumes electricity’ during normal operation and outputs EEG readings. Akin to a PC consuming electricity acting as a side channel in CYB, there is a deterministic correlation between the brain’s internal operations and the output EEG readings. The authors exploited a neurophysiological phenomenon called the Event-Related Potential (ERP), consisting of signatures in EEG readings that correspond to specific visual and auditory stimuli (i.e., a side channel). Participants were exposed to stimuli of possibly known information (e.g., people they know). Output EEGs were analysed via statistical and regression analysis for classification and dimensionality reduction to extract ERP indicating a recognition confirmation. Use of ERP is akin to the *profiling* SCS method, and analysis of the EEG readings are akin to the *machine learning* and *information theoretical analysis* SCS extraction techniques (Section 2.1.7). This example demonstrates the cross-domain transferability of SCS extraction techniques.

It should be recognised that in this approach the domains and target systems are inconsequential. Instead, what is considered are the information parameters (Section 2.1.5). A PC and a brain share comparable physical modalities. Both consume electricity and output usage, while sharing fundamental information parameters of frequency, amplitude, and time. To explore this concept further, we can consider the HCI example of SCS by Low et al. [43]. The output luminance from the flash of a smartphone was quantified using the camera (i.e., the sensor). As the user obstructs the flash, the sensed luminance level varies. This can be parsed as a metric of the ’pressure’ being applied. *Luminance* is the information parameter that describes the target information, which is exploited by their SCS extraction techniques (correlations and regressions). Within the human body, each heartbeat invokes a periodic oscillation of blood volume within the bloodstream. This is a proven side channel for detecting heart rate visually via a camera [28]. When a flashlight is shone onto a person’s skin, the *luminance* of the reflecting light varies in correlation with the volume in the underlying bloodstream, from which the heart rate can be inferred.

Both side channels contain the same information parameters, and require a signal injection (e.g., a flashlight). It is the luminance (the information parameter) that their respective SCS extraction techniques rely on and exploit to operate.

The cases above stem from different target systems and domains, albeit with side channels using comparable information parameters. This view promotes the transferability of SCS extraction techniques. Similarly, the concept applies in cases of intra-domain transferability. For example, given a MDM target system (e.g., the human body), a proven SCS extraction technique for one part of the body may be applicable elsewhere, irrespective of the different physical modalities involved, so long as they share identical information parameters.

Complimenting the transferability hypothesis, we can consider the mindsets underlying each domain. From the ‘attack’ mindset of CYB, involving invasion/modification of the target system, to the desire for non-invasive and even passive sensing of MDM and HCI. Understood is that while covert sensing may not be essential, unobtrusive sensing methods can still be advantageous, particularly within MDM and HCI where non-invasive sensing is preferred. Similarly, adoption of HCI’s willingness to experiment with prototypes and considered sensors may bode well for other domains, particularly due to the propensity for unorthodox sensors. This encourages exploration of potentially existing side channels or the information parameters of quantifiable signals. Shared mindsets include the ability to achieve SCS despite sensor availability constraints, and perhaps the inability to invasively modify or probe target systems. Instead, there is reliance on understanding of side-channel properties and information parameters, along with the adoption of SCS methods and extraction techniques not normally (or at all) associated with a particular domain.

Challenges arise in the sheer diversity in target systems across varying domains. On the surface, viewing a smartphone as akin to the human body, or a PC as akin to an electrical grid appears nonsensical. Furthermore, side channels that share comparable or identical information parameters may not qualify for transferable SCS extraction techniques. Signal noise, diversity in target information, and the SCS methods applicable for the target system (e.g., invasive vs. passive) all influence the approach to SCS. Nonetheless, the SCS framework enables a fundamental and abstracted view of side channels, shedding the constraints of bespoke approaches for specific target systems or domains by providing deeper descriptors of side channels beyond just their physical modality (e.g., electricity, vibration).

### 3.2. Representation of Side Channels

Opportunities exist in parsing the SCS framework into sufficient data structures that can be used computationally. CYB models target systems via data structures such as flow trees and directed graphs. However, they are designed especially for cyber-physical target systems [9,10]. The SCS framework provides a domain-agnostic representation applicable to all target systems. Granted each SCS component is sufficient encoded, a data structure can model the real-world side channels of any target system. Such data structures can then be processed computationally. Within CYB, side channels within target systems are modelled and methodologies developed to detect side-channel leakage for privacy concerns [101] or discover new side channels for novel sensing opportunities [102]. Representations of side channels for MDM, HCI, and Misc are non-existent, as SCS is not yet formally established in these domains. However, the SCS framework demonstrates that despite target system diversity, they can fundamentally be described by the SCS framework components. Consequently, their respective target systems can also be captured as a data structure.

We propose that side-channel *leakage detection* and side-channel *discovery* through modelling of target systems is equally applicable to MDM, HCI, and Misc. Such methods need only look for *determinism* within modelled target systems (Section 2.1.4). The SCS framework enables a formalised and systematic approach to studying side channels, detecting their presence, and exploiting them. Candidate side channels can be studied as per their side-channel properties to understand the connection indicated by the determinism. A suitable SCS method is determined by the defined constraints and intentions (Section 2.1.7). For instance, certain SCS solutions care only for acquiring target information, and can be invasive or local. An effective SCS extraction technique is best identified by study of the information parameters present, or by whether an identical side channel has been proven elsewhere (Section 2.1.5).

For example, the human body is a rich source of side channels (Section 2.1.2). Modelling its structure and their connections to develop MDM solutions is well established. However, a data structure model following the SCS framework is a novel approach. The benefits include viewing the human body as per its side-channel properties and information parameters. Such a data structure can be analysed computationally, akin to how CYB’s target systems are. Instances of determinism point to candidate novel side channels, each describable by the SCS framework.

Consequently, the hypothesis regarding transferability of SCS extraction techniques is directly interlinked (Section 3.1). Side channels discovered via side-channel discovery methods can be analyzed and understood as per the SCS framework. Crucially, the information parameters can be identified. Thus, SCS extraction techniques shown to utilise the identified information parameters are applicable to newly discovered side channels as well.

### 3.3. Additional Domains

The reviewed domains of CYB, MDM, HCI, and Misc represent the most explicit examples of SCS within the literature. However, Misc alludes to other domains that could be named if enough examples are amassed. One example is civil infrastructure and its related target systems, such as electrical grids and road networks. Another potential field involves the environment, both natural and built. Examples include detecting movement behind room walls via WiFi and unique exploitations of benign physical objects, such as detecting audio from the vibrations of chip packets. The SCS framework remains applicable to the target systems of these additional domains irrespective of their structure or components, as all target systems are viewed abstractly. Each generates a cacophony of observable auxiliary signals as a byproduct of their normal operation. Where determinism is confirmed, side channels are suspected, which can be framed by their side-channel properties and understood via the information parameters demonstrated in this review. When these are quantified, appropriate SCS extraction techniques can be employed.

## 4. Conclusions

SCS involves the utilisation of available sensor data in a non-trivial way to acquire previously unknown, hidden, or unused target information from target systems. This review analyses the applicability of the SCS process across four distinct domains. We are the first to establish SCS within the HCI and Misc domains, which hitherto have been ad hoc and isolated. This novel approach broadens the field of SCS beyond the current boundary of MDM and CYB and their respective target systems (Section 2). Despite their diversity, each of these domains consists of a plethora of SCS-related solutions. We have demonstrated that each fundamentally relies on the strong correlation between externally sensed signals and the internal operations of their target system, i.e., the side-channel property of *determinism* (Section 2.1.4). Established fields of advanced sensing and signal processing techniques that exploit this correlation underlie each SCS example across each domain. Irrespective of domain, there is a mindset that noise is potentially the *source* of side channels. SCS can be achieved despite sensor availability constraints through non-obvious and indirect means.

This work provides a theorised unified view of these otherwise isolated domains. We establish that the SCS process can be defined by the distinct yet connected components of target information, target systems, side channels, side-channel properties, information parameters, sensors, and SCS methods and extraction techniques (Section 2). Combined, these form a SCS framework providing a unified, abstract, and domain-agnostic definition of SCS irrespective of the specific environment and application. This framework sufficiently describes the entire SCS process, with the SCS framework components corresponding to real-world observations.

The SCS framework enables a formalised and *systematic* approach to studying side channels, detecting their presence, and exploiting them both within and between domains. As each domain abides by the same SCS framework components, a hypothesis around the intra-domain and cross-domain transferability of SCS extraction techniques emerges (Section 3.1). Demonstrated SCS extraction techniques are applicable to other side channels that exhibit identical information parameters, irrespective of the domain or target system. Consequently, opportunities exist where exploiting the mindset of one domain may be lead to novel outcomes in another. From the ‘attack’ mindset of CYB, willing to invade/modify the target system, to the desire for non-invasive and even passive sensing in MDM and HCI. Techniques and approaches can be transferred between and within domains that subscribe to the SCS framework.

Additional avenues exist in the capture of SCS examples as data structures for computation. A suitable data structure can be representative of real-world SCS examples as well as of a real-world target system. Methodologies can take such data structures as input for side-channel leakage detection and side-channel discovery. These methodologies would inherently be domain-agnostic and applicable to all target systems that can be modelled by the SCS framework (Section 3.2). Lastly, the Misc domain hints at additional domains that could be incorporated into the SCS framework in the future (Section 3.3).

## Figures and Tables

**Table 1:** Terminology for defining the components of the SCS process [12].

Existing Classifications Related to Side-Channel Use	Classifications Used in This Research
Side-channel attacks (relating to side-channel exploitation within CYB only) [14], side-channel sensing (relating to side-channel exploitation within CYB as well as other domains) [2].	Side-channel sensing
Target information (used within MDM and CYB [2] but also applicable to all domains).	Target information
Cryptographic devices (attacks against cyber–physical devices) [14], target systems (within context of MDM and CYB) [1,2].	Target system
Biomarkers (objectively measured characteristics that lead to a diagnosis), side channel (a pathway in which target information traverses along within any context) [1,2,14].	Side-channel
Side-channel properties [2].	Side-channel properties
Modalities (a description of the target information and the signal within which it is embedded) [1].	Information parameters
Measurement setup [14], sensors [2].	Sensors
Leakage models (understanding of the signal within a side channel in a CYB context) [14], techniques [1,15], physical attacks [14], side-channel attack techniques [2].	Methods and Extraction techniques

## Data Availability

No new data were created or analyzed in this study. Data sharing is not applicable to this article.
